# Sperm-borne miR-449b influences cleavage, epigenetic reprogramming and apoptosis of SCNT embryos in bovine

**DOI:** 10.1038/s41598-017-13899-8

**Published:** 2017-10-17

**Authors:** Mengyun Wang, Yang Gao, Pengxiang Qu, Suzhu Qing, Fang Qiao, Yong Zhang, Jesse Mager, Yongsheng Wang

**Affiliations:** 1 College of Veterinary Medicine, Northwest A&F University, Key Laboratory of Animal Biotechnology of the Ministry of Agriculture, Yangling, Shaanxi Province 712100 PR China; 2 Maternity & Child Health Care Hospital, Baoji, Shaanxi Province 721000 PR China; 3 Department of Veterinary and Animal Sciences, University of Massachusetts, Amherst, MA 01003 USA

## Abstract

Accumulating evidence indicates the absence of paternally derived miRNAs, piwiRNAs, and proteins may be one important factor contributing to developmental failure in somatic cell cloned embryos. In the present study, we found microRNA-449b (miR-449b) was highly expressed in sperm. Target gene predictions and experimental verification indicate that several embryonic development-related genes, including *CDK6*, *c-MYC*, *HDAC1* and *BCL-2*, are targets of miR-449b. We therefore investigated the role of miR-449b using somatic cell nuclear transfer (SCNT) embryo model. Bovine fetal fibroblasts, expressing miR-449b through a doxycycline (dox) induced expression system were used as nuclear donor cells for SCNT. The results showed that miR-449b expression in SCNT embryos significantly enhanced the cleavage rate at 48 h after activation and the levels of H3K9 acetylation at the 2-cell to 8-cell stages, meanwhile, significantly decreased the apoptosis index of blastocysts. In addition, we verified miR-449b could regulate the expression levels of *CDK6*, *c-MYC*, *HDAC1* and *BCL-2*. In conclusion, the present study shows that miR-449b expression improves the first cleavage division, epigenetic reprogramming and apoptotic status of bovine preimplantation cloned embryos.

## Introduction

Somatic cell nuclear transfer (SCNT) is a promising technology for both livestock production and human therapeutic cloning towards tissue and organ engineering. However, SCNT efficiency remains very low, with normal offspring rates less than 5% in most species^1^, which is a major hindrance for wide application of the promising technique. A variety of evidence indicates that incomplete epigenetic reprogramming of the donor nucleus is an important reason causing developmental failure of SCNT embryos^2,3^. Aberrant epigenetic modifications, including genomic DNA methylation, histone modifications, and miRNA misregulation, have been observed at every developmental stage of SCNT embryos^4–6^. Therefore, improving reprogramming of the donor nucleus may be the key to enhance the efficiency of SCNT.

Traditional views regard the main function of sperm is to deliver the paternal haploid genome to the oocyte and activate development of a new life after fertilization, while the main function of the oocyte is to provide the maternal haploid genome and various important factors for the development of embryo. As a result, researches identifying regulatory factors related to epigenetic reprogramming and embryonic development have been largely focused on oocytes^7,8^. However, recent studies have shown that sperm delivers not only paternal haploid chromosomes but also many factors including proteins, mRNAs, tRNAs, piwiRNAs, and miRNAs^9,10^. Moreover, these paternal regulatory factors play important roles in the formation of male and female pronucleus, temporal order of blastomere division, zygotic genome activation, development of embryos, and even the phenotype of offspring^11,12^. Liu *et al*. confirmed that sperm-born miR-34c has a critical effect on the first cleavage after fertilization - all embryos were blocked at the 2-cell stage after miRNA-34c inhibitor treatment^13^. These findings indicate that the complete lack of sperm-born regulatory factors might be a major contribution to developmental failure of SCNT embryos.

miRNAs are small 22 to 24 nucleotide non-coding RNAs that play important roles in post-transcriptional regulation of gene expression in multicellular organisms by affecting both the stability and translation of mRNAs through the RNA-induced silencing complex (RISC). Through this post transcriptional regulation, miRNA can influence gene translation of many genes and thus regulate various biological processes. miR-449b is a member of miR-449 cluster composed of miR-449a, miR-449b, miR-449c and miR-449d. Gene ontology (GO) annotations indicate that miR-449 related pathways include DNA damage response, cell cycle, and senescence and autophagy in cancer. Previous studies about the function of miR-449b mainly focused on its regulatory role in the progression of cancer^14–16^. Kheir *et al*. showed a loss of miR-449 expression in human gastric tumours compared to normal tissues^17^. Yang *et al*. indicated that miR-449a/b suppresses tumor progression through regulating Rb/E2F1 activity, and escape from this regulation through aberrant epigenetic events contributes to E2F1 deregulation and unrestricted proliferation in human cancer^18^. In our previous studies we found that miR-449b was highly expressed in bovine sperm^19^, suggesting that it might be delivered into oocytes and participate the development of embryos after fertilization.

Target gene predictions and relative researches indicated that *BCL-2*, *CDK6*, *c-MYC*, *HDAC1*, *NANOG* and *CCND1* might be the target genes of miR-449b. Therefore, we speculated that miR-449b delivered in the sperm might improve the acetylation level of histone by down-regulating the expression of *HDAC1* during embryonic development and promote epigenetic reprogramming. Several studies have indicated that miR-449b may be involved in histone deacetylation; Buurman *et al*. reported that up-regulation of HDAC1-3 reduces expression of miR-449 in hepatocellular carcinoma cells^20^, and William *et al*. observed that the influenza-induced expression of miR-449b interacted with the histone deacetylase HDAC1 to alter IFN-β gene expression^21^. In addition to *HDAC1*, we checked the functions and pathways of other genes on NCBI, which were involved in cell cycle and apoptosis. We noticed that during early embryonic development in bovine, miR-449b related studies were few, so we attempted to explore whether miR-449b plays a regulatory role in bovine embryonic development.

Systems for spatial and temporal manipulation of gene expression are essential tools for developmental studies and are of particular importance for research in gene or miRNA function in certain developmental stages or tissues^22^. The Tet-On system, which consists of response element (pTRE3G-BI) and regulatory element (pEF1 α-Tet3G), developed from the Tet-off system constructed by Gossen and Bujard in 1995, can induce the expression of target gene by tetracycline or its derivatives. A low dose of doxycycline (Dox) regulates the expression of target gene without toxicity to animals or cells. Numerous studies have shown inducible Tet-On system is a convenient tool for reversible and very tightly controlled conditional gene expression in zebrafish, drosophila, murine, porcine and bovine models^23–27^. In our previous study, we successfully used the Tet-On system to induce expression of miR-34c in bovine fibroblasts^28^.

The dual luciferase reporter system is a method to detect the activity of firefly luciferase with luciferin as substrate. Luciferase can catalyze the oxidation of fluorescein and produce biologically fluorescent. The 3′UTR of potential target genes, which may be complementary to relative miRNA, could be cloned to the MCS downstream of the stop codon of hRluc (renilla luciferase) gene on pRCHECK^TM^-2 vector. If miRNA interacts with the 3′UTR of any target gene in a completely complementary or incomplete form, the corresponding degradation or inhibition for translation of the mRNA downstream of the T7 promoter will lead to decreased activity of hRluc. In addition, psiCHECK^TM^-2 vector can simultaneously express hRluc and the hluc+ (firely luciferase) in the cell, and both of them have no provenance homology and correspond to different reaction substrates without cross-interference, so the hluc+ downstream of TK promoter was regarded as an internal control to normalize the fluorescence intensity of hRluc.

In the present study, the effects of miR-449b on the development of embryos was investigated using SCNT embryo as a model. Bovine fetal fibroblasts, expressing similar level of miR-449b as sperm through the control of Tet-On system, were used as nuclear donor cells for SCNT. We monitored the development of SCNT embryos and the acetylation level of histone H3K9 (H3K9ac) in different stages (2-cell, 8-cell, and blastocyst) by immunofluorescence. The relative expression levels of miR-449b target genes were examined at the 2-cell and 8-cell stages by qRT-PCR. Additionally, the terminal deoxynucleotidyl transferase mediated deoxyuridine triphosphate nick end labeling (TUNEL) assay was performed on embryos to determine the apoptotic index of embryos.

## Results

### Expression of miR-449b in oocytes, fibroblasts, sperm, dox-induced cells, and embryos derived from IVF, SCNT, and PA

**Figure 1:**
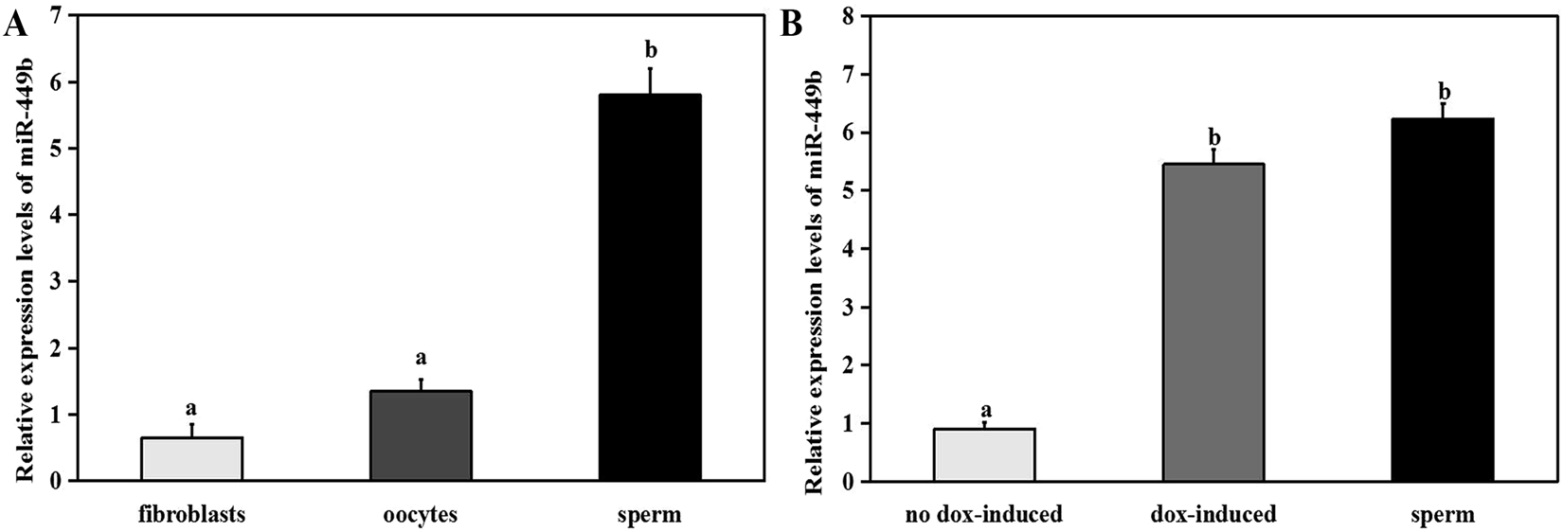
Quantitative (q) PCR analysis of relative expression levels of miR-449b in fibroblasts, oocytes, and sperm (**A**), as well as in no dox-induced fibroblasts, dox-induced fibroblasts, and sperm (**B**). The real-time data were normalized to the expression of the small nuclear RNA U6 and analyzed by v.2.1 StepOne Software. Comparisons between experimental groups were performed using the ΔCt method (ΔCt = Raw Ct (miRNA) - Raw Ct (U6)), where fold change was expressed as 2^−ΔCt^. The bars graphs indicate the mean ± SEM from three independent biological samples. a,b Values with different superscripts within columns are significantly different from each other (P < 0.05).

Total RNA was successfully isolated from sperm, oocytes, and fetal fibroblast cells using different methods. Though weak expression of miR-449b was discovered in oocytes and fibroblasts, expression levels were significantly higher in sperm (Fig. 1A).

**Figure 2:**
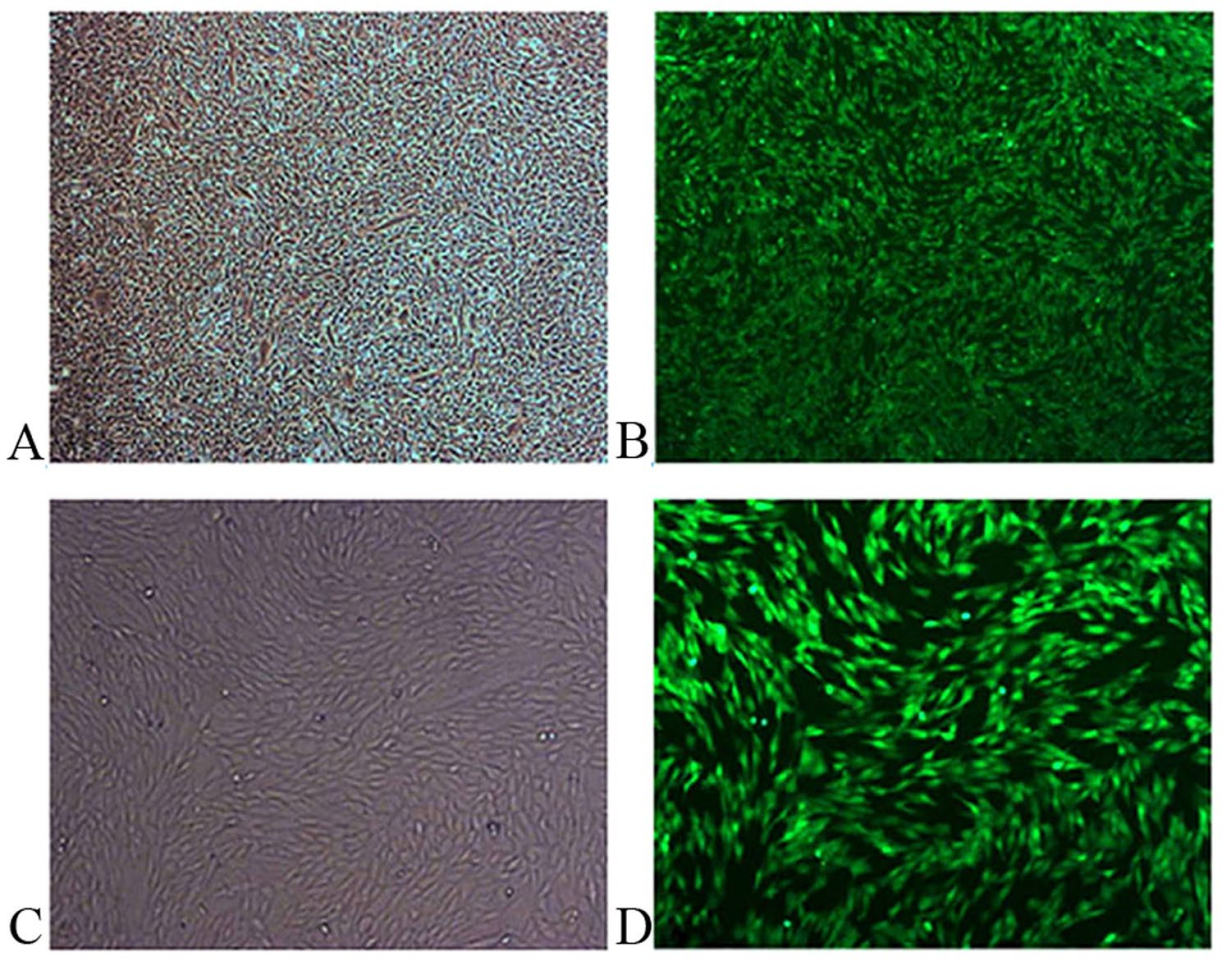
Representative images of G418-resistant colonies after transfection with Tet-On 3 G expression vector (miR-449b-pTRE3G-BI-eGFP and pEF1α-Tet3G). (**A**) Magnification 40×, shows the bright-field view of (**B**); (**C**) magnification 100×, shows the bright-field view of (**D**).

After co-transfection of bovine fetal fibroblast cells with the response element (miR-449b-pTRE3G-BI-eGFP) and regulatory element (pEF1α-Tet3G), positive clones were selected using G418 for more than one week, and the fluorescence intensity and the growth state of cells were observed by fluorescence microscopy. It is worth noting that green fluorescence can be detected only in the dox-induced cells, while not in dox-induced cells (Fig. 2A–D). Then, the expression levels of miR-449b was examined in dox-induced cells, non-induced cells and sperm, and these data were normalized to the expression of small nuclear RNA U6. The Results showed that the relative expression level of miR-449b in dox-induced cells was similar to sperm, and significantly higher than that in no dox-induced cells (Fig. 1B).

Meanwhile, we characterized the expression of miR-449b in 1-cell, 2-cell, 8-cell embryos derived from *in vitro* fertilization (IVF), SCNT, and parthenogenetic activation (PA). As shown in the result, the expression of miR-449b was similar in the IVF and NT-miR449b group, but significantly higher at the 1-cell stage, indicating that induced miR-449b level in NT could be comparable to IVF zygotes, meanwhile, sperm-borne miR-449b did not degrade immediately after fertilization but remained in zygotes. The miR-449b expression among three groups decreased at the 2-cell stage and significantly lower in the NT-control group, however, increased significantly in IVF group at the 8-cell stage compared to the NT-control and NT-miR449b group (see Supplementary Fig. S1). Compared to PA embryos, we observed that miR-449b was more strongly expressed in IVF during early-development stage, indicating that we could exclude the interference from maternally and zygotically (see Supplementary Fig. S2). Besides, the expression pattern of miRNA-449b in bovine IVF preimplantation embryos can be found as Supplementary Fig. S3.

### miR-449b overexpression prolonged the first cleavage time of SCNT embryo

**Figure 3:**
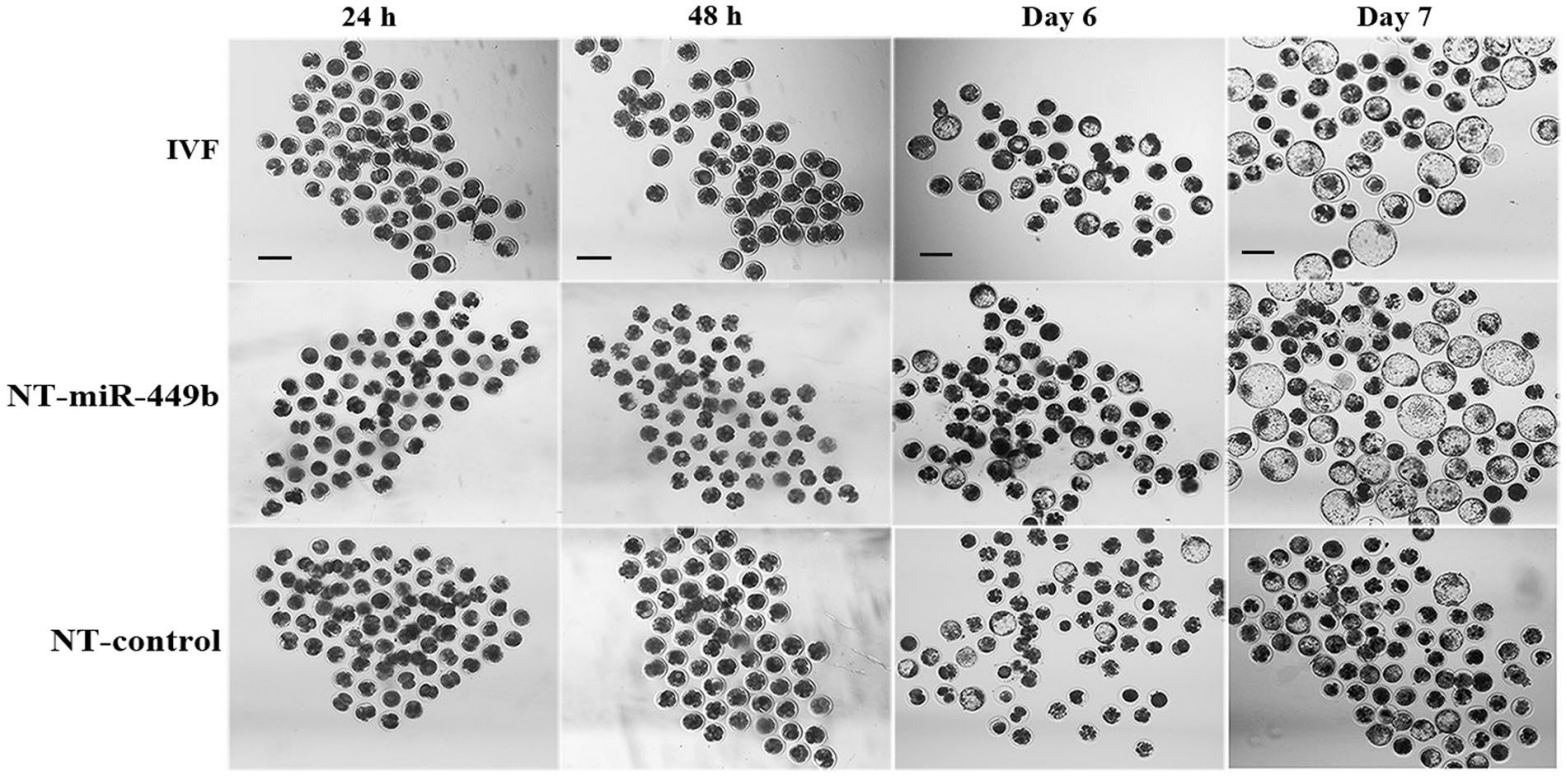
Representative photographs of bovine embryo. Development of SCNT embryos in the IVF, NT-miR-449b, and NT-control groups at 24 h, 48 h, day 6 and day 7 after activation/insemination were showed in corresponding image, respectively. Scale bar 200 μm.

Since the main function of two target genes, *CDK6* and c-*MYC*, involved in cell cycle regulation, we examined the speed of embryonic development as measured by timing to cleavage divisions. As shown in Table 1 and Fig. 3, the cleavage of the NT-control embryos was faster than IVF embryos at 24 h after activation or insemination, i.e. more than a half of NT-control embryos were 2-cell while the cleavage rate is only 35.47% ± 1.96 and 37.68% ± 2.14 in NT-miR-449b and IVF group respectively, which is significantly lower than that of NT-control group (P < 0.05). However, at 48 h, the cleavage rate was significantly higher in the NT-miR-449b and IVF than NT-control group (78.02% ± 1.34, and 69.28% ± 2.54 vs 61.50% ± 3.21, P < 0.05). These results indicate that miR-449b influences the first cleavage division. Although, no significant difference in blastocyst formation rate on day 7 was observed among the three groups (30.36% ± 2.02 and 30.19% ± 1.67 vs 22.18% ± 2.34, P > 0.05), blastocyst formation rate on day 6 was significantly higher in the NT-miR-449b and IVF than NT-control group (30.19% ± 1.37 and 29.36% ± 2.02 vs 18.87% ± 2.34, P < 0.05).

**Table 1:** Effect of three groups on developmental competence of bovine SCNT embryos.

Group	No.embryos cultured (3 replicates)	No.(%) cleavage rate at 24 h	No.(%) final cleavage rate at 48 h	No.(%) blastocysts on day 6	No.(%) blastocysts on day 7
IVF group	165	39(37.68 ± 2.14)^a^	114(69.28% ± 2.54)^a^	48(29.36% ± 2.02)^a^	50(30.19% ± 1.67)^a^
experimental group	158	56(35.47 ± 1.96)^a^	123(78.02% ± 1.34)^a^	48(30.19% ± 1.37)^a^	49(30.36% ± 2.02)^a^
control group	155	79(51.20% ± 3.21)^b^	95(61.50% ± 3.21)^b^	29(18.87% ± 2.34)^b^	34(22.18% ± 2.34)^a^

Three replicates were performed. Numbers in parentheses (mean ± SEM%) represent development rates (2-cell at 24 h, 4-cell at 48 h and blastocysts on day 6 and day 7), while other numbers indicate total embryo numbers of three replicates. ^a,b^values with different superscripts within columns are significantly different from each other (P < 0.05).

### miR-449b overexpression increased global histone acetylation levels of SCNT early embryos

**Figure 4:**
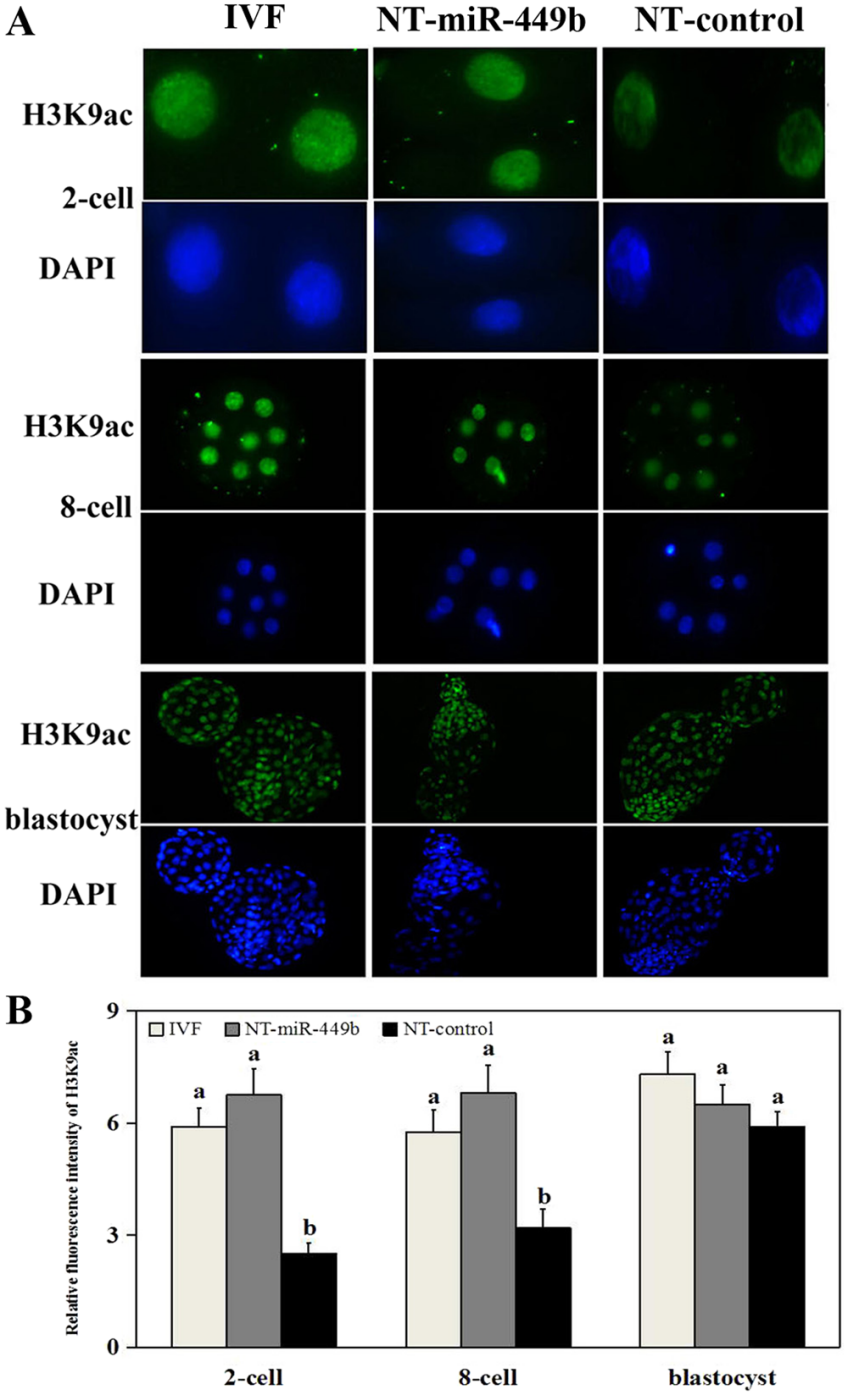
Immunofluorescence analysis of H3K9ac in various bovine embryos. (**A**) Global acetylation level of H3K9 (green) in embryos at the 2-cell, 8-cell, and blastocyst stages in IVF, NT-control, and NT-miR-449b groups. Each sample was counterstained with DAPI to visualize DNA (blue). Original magnification, 200×. (**B**) Relative fluorescence intensities of H3K9ac (using Image-Pro 6.0 software, and the values were showed as mean ± SEM). a,b Values with different superscripts within columns are significantly different from each other (P < 0.05).

2-cell, 8-cell, and blastocysts were collected for H3K9ac immunofluorescence staining. In the IVF and NT-miR-449b groups, the acetylation levels of histone H3K9 were significantly higher at the 2-cell and 8-cell stages than that of the NT-control group, and there was no difference between the NT-miR-449b and IVF embryos. While at the blastocyst stage, no significant differences in acetylation levels of histone H3K9 were detected among the three groups (Fig. 4).

### miR-449b overexpression decreased apoptosis index of SCNT embryos

**Figure 5:**
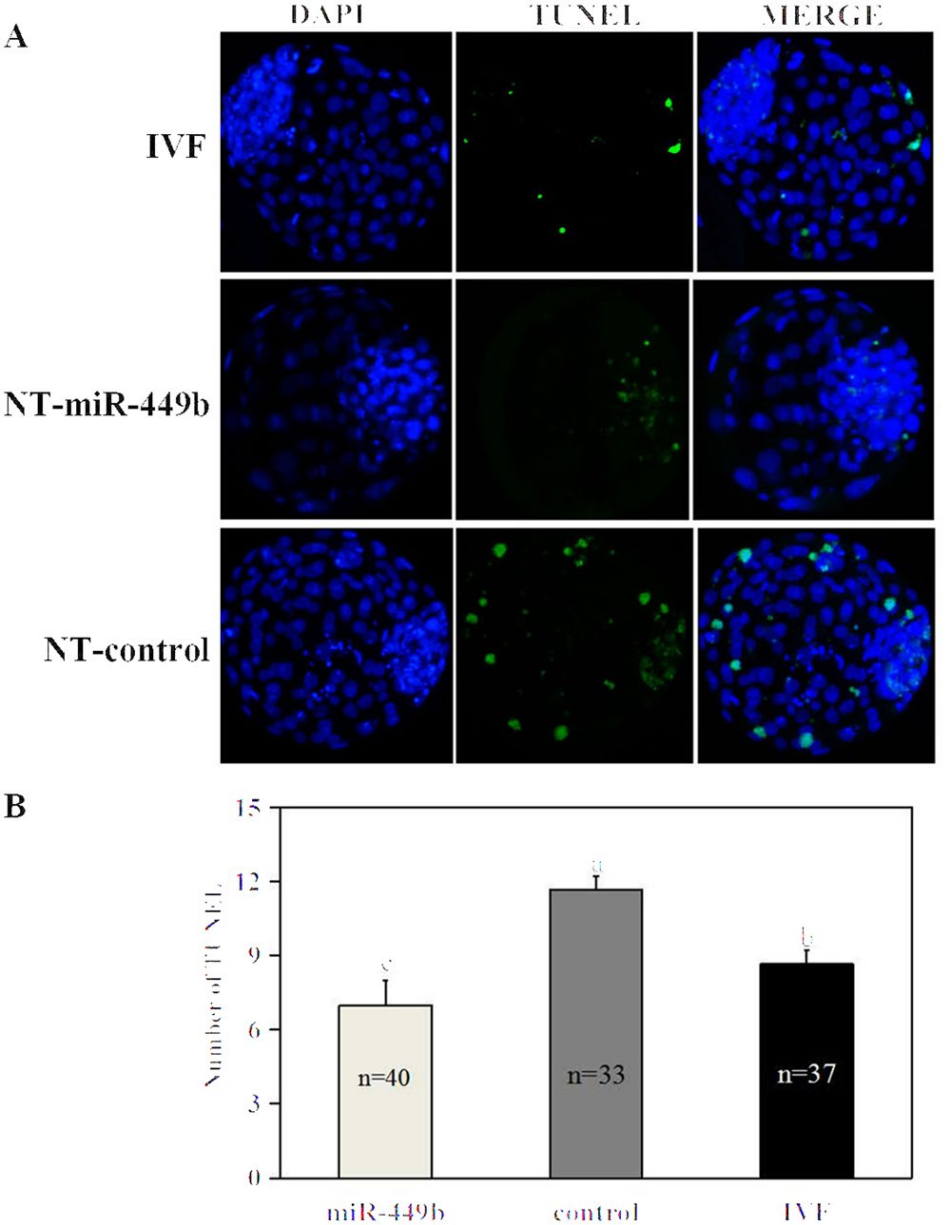
Incidence of apoptosis in blastocysts. (**A**) TUNEL assay of blastocysts (green). Each sample was counterstained with DAPI to visualize DNA (blue). Original magnification, 200×. (**B**) Number of apoptotic cells in each blastocyst. a,b,c Values with different superscripts within columns are significantly different from each other (P < 0.05).

Apoptosis index was examined on day 7 blastocysts using deadend fluorometic TUNEL system. As shown in Fig. 5A, no significant differences in the apoptosis index between the IVF and NT-miR-449b group, while apoptosis index in the NT-control blastocyst was significantly higher than both the IVF and NT-miR-449b groups (P < 0.05). However, the number of apoptotic cells in each blastocyst among three groups was significantly different (Fig. 5B).

### Confirmation of target genes of miR-449b in early embryos

**Figure 6:**
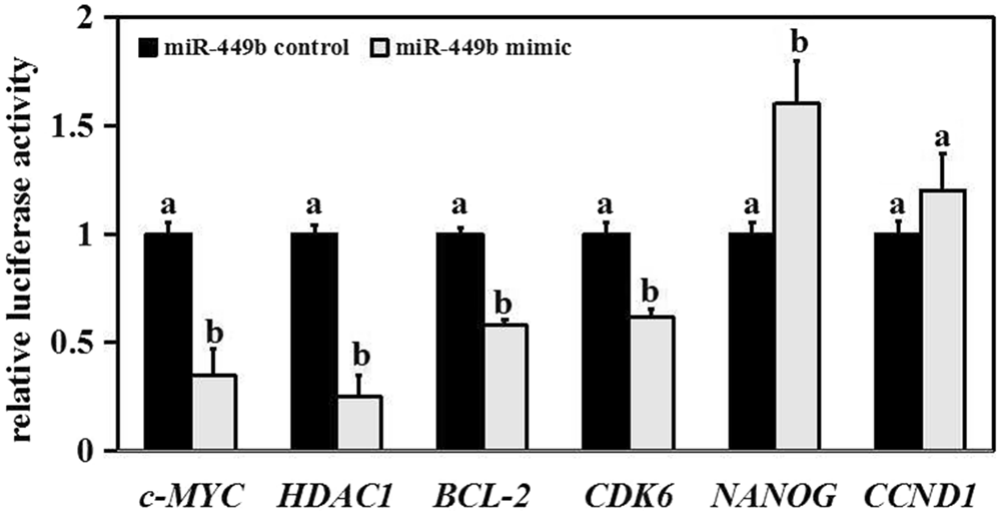
Dual-Luciferase reporter assay. The bars graphs indicate the ratios (renilla luciferase/firely luciferase) of different putative target genes, and the result showed that the ratios of *c-MYC*, *HDAC1*, *BCL-2*, and *CDK6* were reduced when compared with the control group, while *NANOG* and *CCND1* were increased, suggested *c-MYC*, *HDAC1*, *BCL-2*, and *CDK6* might be the target genes of miR-449b *in vivo*. a,b Values with different superscripts within columns are significantly different from each other (P < 0.05).

According to predictions of target genes using the TargetScan and miRanda software and relative literature, *c-MYC*, *HDAC1*, *BCL-2*, *CDK6*, *NANOG* and *CCND1* were selected as candidate targets of miR-449b. The levels of firefly luciferase and renilla luciferase of candidate genes were detected after miR-449b mimic/mimic control and recombinant plasmid psiCHECK^TM^-2-3′UTR were co-transfected into 293T cells. The result showed that the relative luciferase activity (renilla luciferase/firely luciferase) of *c-MYC*, *HDAC1*, *BCL-2*, and *CDK6* was significantly reduced when compared with the control group (Fig. 6), indicating they were regulated by miR-449b *in vivo*. However, the relative luciferase activity of *NANOG* and *CCND1* increased abnormally.

**Figure 7:**
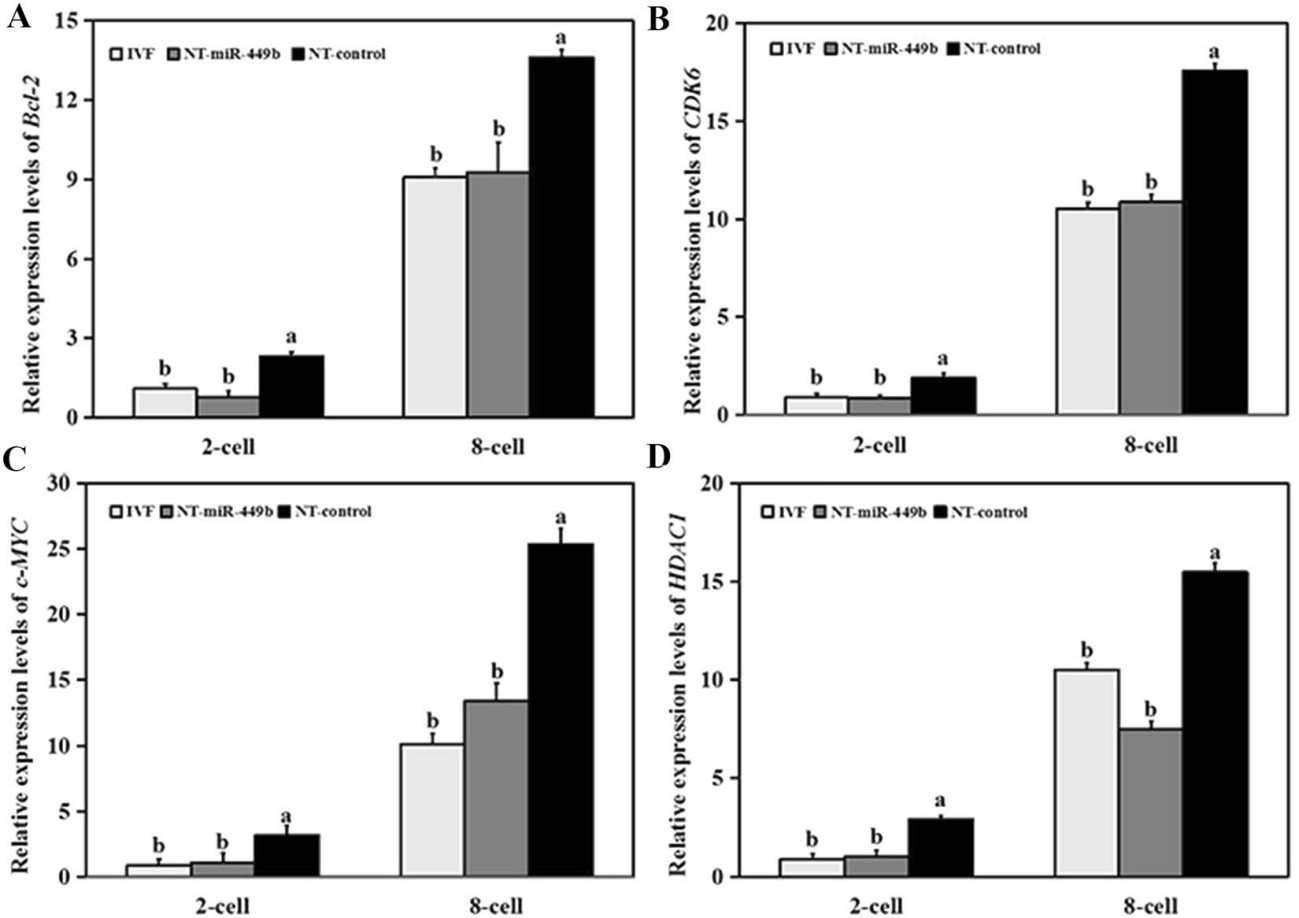
Relative expression levels of the target genes of miR-449b. The expression levels of *BCL-2* (**A**), *CDK6* (**B**), *c-MYC* (**C**), and *HDAC1* (**D**) were analyzed by qPCR, respectively. They were significantly lower in the NT-miR-449b group than NT-control group, but no differences compared with IVF group. The real-time data were normalized to GAPDH and analyzed by v.2.1 StepOne Software. Comparisons between experimental groups were performed using the ΔCt method (ΔCt = Raw Ct (mRNA) - Raw Ct (GAPDH)), where fold change was expressed as 2^−ΔCt^. The bars graphs indicate the mean ± SEM from three independent biological samples. a,b Values with different superscripts within columns are significantly different from each other (P < 0.5).

In addition, the expression levels of these target genes were detected in different developmental stages of preimplantation embryos derived from IVF, NT-miR-449b (dox-induced cells as nuclear donor) and NT-control group (non-induced cells as nuclear donor). The expression levels of *BCL-2*, *CDK6*, *c-MYC*, and *HDAC1* were significantly higher without miR-449b overexpression (NT-control group) at both the 2-cell and 8-cell stages, but returned to the level of IVF group when donor cells were treated with dox (Fig. 7). These results indicated that miR-449b might play pivotal roles in cell cycle regulation (*CDK6* and *c-MYC*), cellular apoptosis (*BCL-2*), and epigenetic reprogramming (*HDAC1*).

## Discussion

Mature sperm is highly specialized and compact cells that carry minimal cytoplasm. Unlike somatic cells, no replication, transcription or translation are thought to occur in sperm. Related studies have demonstrated that the cytoplasm of mature sperm is extraordinarily low, but it still contains some mRNAs, miRNAs, endogenous small interfering RNAs (endo-siRNAs), and piwi-interacting RNAs (piRNAs)^29–33^. The role of these sperm RNAs on the development of embryos is controversial. Traditional view regards that these sperm RNAs are residues of spermatogenesis, and they have no effect on the embryonic development after fertilization. However, some recent studies indicated that the sperm miRNAs play important roles on embryonic development. Yuan S *et al.* found that embryos fertilized by spermatozoa with aberrant miRNAs or/and endo-siRNAs profiles by male germ cell-specific Dicer and Drosha cKO (conditional knock out) mice displayed reduced preimplantation developmental potential. However, preimplantation development could be rescued by addition of wild-type sperm RNAs. These experiments suggested that sperm-borne miRNAs and endo-siRNAs are important for preimplantation embryonic development^10^. In the present study, we found that sperm-born miR-449b played important roles on cleavage timing, blastocyst formation, epigenetic reprogramming, and blastomere apoptosis. Our results further confirm that sperm-borne miRNAs may be widely involves in early developmental evens.

In previous SCNT studies in bovine, we observed that the first cleavage time was shortened in SCNT embryos than that in IVF embryos (unpublished). There are numerous developmental events occur prior to the first cleavage, including syngamy, early embryonic genome activation, and epigenetic reprogramming. In human, Lundin *et al*. confirmed that ICSI embryos had a higher rate of early cleavage than IVF embryos, which was similar to our finding, i.e. the cleavage speed of embryos obtained from IVF was later^34^. Although the results are similar, the corresponding mechanism is different. We speculated the faster cleavage in SCNT mainly due to the deficiency of sperm-borne regulatory factors, such as miRNAs, proteins, mRNAs and tRNAs. In addition, the difference of cleavage speed is related to numerous factors, such as activation, cytoskeleton and gene expression pattern. ICSI is a more direct means of insemination compared with IVF, because it bypasses many steps such as acrosome reaction and zona reaction, etc., which were essential for IVF. In the present study, we found that miR-449b influenced not only cleavage rate but also cleavage speed, i.e. the first cleavage occurred 24 h later in most of IVF and miR449b supplemented SCNT embryos, while for the NT-control embryos, the first cleavage occurred before 24 h after activation/insemination. These results indicated that sperm-born miR449b might be involved in the regulation of the timing of the first cleavage.

Previous study has shown that injection of sperm-borne miR-34c inhibitor into zygotes could inhibit DNA synthesis and significantly suppress the first cleavage division in mouse^13^. In bovine, we also observed that high expression of miR-34c in somatic cell donor cells affected cleavage and development of SCNT embryos^28^. As part of the same family, miR-34c and miR-449b share the same seed sequence and many target genes^14,35^. Therefore, we postulated that sperm-born miR-34c and miR-449b might cooperate in the regulation of embryonic first cleavage. Though these regulatory mechanisms remain unclear, two cell cycle regulatory genes, CDK6 and *c-MYC*, were identified as the target genes of miR-449b. Previous studies showed that miR-449a/b targeted and inhibited oncogenic CDK6 and resulted in the dephosphorylation of pRb and cell cycle arrest at G1 phase^18^. Similarly, *c-MYC* participates in different cellular functions, including the cell cycle, cell survival, protein synthesis, cell adhesion and micro-RNA expression^36^. The growth arrest phenotype did not ensue when the expression of *c-MYC*, a target of the Wnt pathway in colorectal cancer (CRC) cells *in vitro* ^14^ and in normal crypts *in vivo* ^37^, was artificially maintained during Wnt pathway inhibition, suggesting that *c-MYC* played a crucial role downstream of the Wnt cascade in maintaining the proliferative status of CRC cell^38^. However, there is no report about how these two genes regulate the first cleavage in early embryos. Further studies will be required to clarify the underlying mechanism of how these two genes regulate the first embryonic cleavage through sperm-born miR-449b.

As one of the critical factors that influence the reprogramming efficiency of donor nuclei, changes in histone acetylation have attracted extensive attention in both SCNT and iPS research. In eukaryotes, histones include core proteins H2A, H2B, H3, H4, and histone linker H1. Histone acetylation mostly occurs in specific lysine residues of N-terminal tails of the core protein in which enriched basic amino acid. Histone acetyltransferase (HAT) or histone deacetylase (HDAC) add and remove acetyl derived from acetyl coenzyme A (CoA) to the ε-NH3+ of histone and regulate the expression of genes^39^. Increased acetylation may weaken the interaction between histones and the DNA backbone, promoting looser structure of histone-DNA complexes, thereby promoted gene transcription^40^. In addition, there is a close relationship between the quality of SCNT embryos and histone acetylation^41,42^. Some studies have shown that the level of H3K9ac may influence the reprogramming of SCNT and embryonic development^43^. In this study, we found that histone H3K9ac was significantly lower at the 2-cell and 8-cell stages in NT-control embryos, but its expression level could similar to the IVF by miR-449b overexpression in donor cells. The above results indicated that miR-449b may be involved in the reprogramming of histone acetylation that occurs during early post-fertilization events. *HDAC1* (histone deacetylase 1), could restore the positive charge of histone, inhibit the transcription of gene and regulate gene expression. In the present study, *HDAC1* was verified as one of the important target genes of miR-449b, suggesting that miR-449b delivered in the sperm might improve the acetylation level of histone by down-regulating the expression of *HDAC1* during embryonic development and promote the reprogramming.

Although miR-449b mimic could alter the expression level of miR-449b in cells, it did not exclude other endogenous miRNAs (regulate *NANOG* or/and *CCND1*) or CeRNA (binding miR-449b competitively). We inferred that these potential RNAs in cells may be involved in the regulatory network of miR-449b through a particular way. Therefore, the fluorescence intensity of *NANOG* and *CCND1* in dual luciferase reporter system was increased abnormally. Besides, the intracellular environment also affected the temporal regulation of miR-449b.

In summary, the present results indicated that sperm-borne miR-449b may play crucial roles in early developmental regulation, specifically prior to the first cleavage division. These results lay the foundation for further exploring the abnormal molecular mechanism occured after nuclear transfer and improving the efficiency of SCNT.

## Methods

All procedures were approved by the Animal Care and Use Committee of Northwest A & F University and performed in accordance with animal welfare and ethics. Except where otherwise noted, all chemicals were purchased from Sigma-Aldrich (St. Louis, USA).

### Sperm purification

Purification of sperm was performed using standard techniques^44^. Frozen-thawed sperm were gently transferred to the bottom of a 15-mL tube with 5 mL of Brackett and Oliphant (BO) medium supplemented with 6 mg/mL BSA and 20 μg/mL heparin, and incubated at least 30 min in a humid atmosphere of 5% CO_2_ at 38.5 °C. The suspensions were collected in a new tube and centrifuged at 1000 × g for 10 min. Then, the supernatants was discarded and the precipitation at the bottom was sperm.

### Oocyte collection and maturation *in vitro*

The methods of oocyte collection and *in vitro* maturation (IVM) were based on previous description^44^. Follicular fluid was aspirated from surface-visible follicles with diameters between 2 and 8 mm using 12-gauge needle attached to a 10-mL syringe. Cumulus oocyte complexes (COCs) with at least 3 layers of cumulus cells and uniform cytoplasm were selected. After washing several times in PBS supplemented with 5% (v/v) FBS, the COCs were cultured 20 h in Tissue Culture Medium-199 (TCM-199, Gibco, America) containing 10% (v/v) FBS, 1 mg/mL 17β-estradiol, and 0.075 IU/mL human menopausal gonadotropin (HMG) in humid atmosphere of 5% CO_2_ at 38.5 °C.

### Construction of Tet-On 3G expression vector

The pre-miR-449b sequence derived from miRBase (http://www.mirbase.org/) was artificially synthesized and ligated into the PUC57 vector by GenScript Co. (Nanjing, China). To obtain the pre-miR-449b and the eGFP (enhanced green fluorescent protein) fragments, double enzyme digestion of the Kpn I-Pst I site of PUC57 and the BamH I-Not site of pd1EGFP-N1 (Clontech, Mountain View, CA, USA) was performed, respectively. The pre-miR-449b and eGFP fragments were inserted into the multiple cloning site (MCS) of the pTRE3G-BI vector and yielded the final construct (miR-449b-pTRE3G-BI-eGFP). After agarose gel electrophoresis and isolation, the plasmid DNA was sequenced and then purified for cell transfection.

### Culture and transfection of bovine fetal fibroblast cells

Bovine fetal fibroblast cells were cultured as described previously^28^. The head and viscera of 35-day-old fetuse were discarded. The remaining tissues were washed thrice with PBS, cut into small pieces (approximately 1 mm^3^). Explants were incubated on 60-mm culture dish with Dulbecco’s Modified Eagle’s Medium (DMEM, Gibco) supplemented with 10% FBS, 100 U of penicillin, 10 ng/mL epidermal growth factor and 250 ng of amphotericin B in a humid atmosphere of 5% CO_2_ at 38.5 °C. After 24 h, 3 mL fresh culture medium was added into dish. When cells had reached 70–80% confluency, cells was passaged by treating with TE (0.25% trypsin/0.05% EDTA). Some cells were frozen in 50% FBS, 40% medium, and 10% DMSO (Sigma) for future use and long-term storage. When needed, cells were thawed and grown in DMEM/F12 (Gibco) medium containing 15% FBS and incubated in a humid atmosphere of 5% CO_2_ at 38.5 °C.

The transfection of Tet-On 3G expression vector into cells was performed according to previous description^28^. Briefly, cells were transfered into 4-mm cuvette gap with 10 μg of miR-449b-pTRE3G-BI-eGFP and 5 μg of pEF1α-Tet3G, and transfected using the BTX Electro Cell Manipulator ECM2001 with three pulses of 1-msec at 510 V. Then transfected cells were transferred to a 90-mm dish for culture. Fresh medium was added after 8 h. Resistant colonies were selected with G418 (Gibco, America) after 48 h. Cells derived from same G418-resistant clone were placed into 6-well plate and treated with or without dox (1000 ng/mL) to induce the expression of miR-499b or no expression according to the experimental design.

### Prediction and identification of target genes for miR-449b

Target genes for miR-449b were predicted using the TargetScan and miRanda software, and the intersection of target genes were selected. Gene sequences from NCBI confirmed seed sequence of miR-449b in the 3′UTR of putative target genes, and those with 3′ UTR matches were further considered as targets.

3′UTR sequence of candidate genes (derived from NCBI) were amplified with primers with Pme I and Xho I sites using the cDNA obtained from bovine fibroblast cells as the template, and inserted downstream of the stop codon of hRluc gene on psiCHECK^TM^-2 vector (Promega, USA). The final vectors (3′UTR constructs) were purified followed by sequencing, and the rest plasmid for future cell transfection. Next, miR-449b mimic/mimic control and recombinant plasmid psiCHECK^TM^-2–3′ UTR were co-transfected into 293T cells. Cells were cleaved after 36 h for dual luciferase reporter assay. The dual luciferase reporter assay was performed using TransDetect Double-Luciferase Reporter Assay Kit (TransGen Biotech, China) based on the manufacturer’s procedure. Briefly, we mixed 100 μL Luciferase Reaction Reagent and 20 μL cell lysates, and detected the firefly luciferase activity using VICTOR^TM^ x5 Multilabel Plate Reader (PerkinElmer, USA). Then, we added 100 μL Luciferase Reaction Reagent II to above mixtures and mixed carefully, followed by the renilla luciferase activity detection. Results were presented as relative luciferase activity (renilla luciferase/firefly luciferase).

### Total RNA isolation and reverse transcription

For isolation of sperm RNA, 500–600 μL Trizol (Invitrogen, USA) was added in the sedimentation of sperm, and total RNA was isolated based on the manufacturer’s instruction with some modification.

The bovine fetal fibroblast cells were washed twice with PBS, and trypsinized with 0.25% TE. Equal volume of DMEM medium containing 10% FBS was added once cells became rounded. Suspensions of cell were removed to a new RNase-free tube and centrifuged at 1000 × g for 5 min to discard the supernatant. Finally, we added 1 mL of Trizol heated in advance to isolate total RNA of the bovine fetal fibroblast cells based on the manufacturer’s instruction.

After IVM for 20 h, the COCs were treated with 0.1% bovine testicular hyaluronidase to disperse their cumulus cells. Oocytes with the first polar (metaphase II) were selected using microscopy. According to the manufacturer’s instruction, oocytes were lysed with the Cells-to-Signal Lysis Buffer (Thermo Fisher, USA).

The reaction of RNA reverse transcription was performed using miScript II RT Kit (Qiagen, Germany) based on the manufacturer’s procedure. Briefly, we mixed 4 μL of 5× miScript Hispec Buffer, 2 μL of 10× miScript Nucleics Mix, 2 μL miScript Reverse Transcriptase Mix, 10 μL purified RNA and 2 μL of RNase-free water for each reaction for a total volume of 20 μL. Thermal-cycling conditions were as follows: 37 °C for 60 min and 95 °C for 5 min.

### Quantitative real-time PCR

The expression level of individual gene and miRNA was detected by SYBR Premix ExTaq^TM^ II (TAKARA, Japan). The mature sequence of miR-449b, U6 and corresponding gene were derived from miRBase and NCBI, respectively, and primers were synthesized by AuGCT DNA-SYN Biotechnology Company (Beijing, China; Table 2). 5 μL of 2 × SYBR Green II, 0.2 μL of 50 × Rox dye, 1 uL of template and 3 μL of RNase-free water were mixed. The PCR cycle conditions were as follows: 95 °C for 30 sec followed by 40 cycles of 95 °C for 5 sec and 60 °C for 30 sec.

**Table 2:** Primers for qRT-PCR.

Name	Primer sequence (5′-3′)	GenBank accession number
miR-449b*	F: GGGAGGCAGTGTATTGTTAGCTG	—
U6*	F: CGCTTCGGCAGCACATATACTA	—
*CDK6*	F: CCTGGACTTTCTTCATTCTCACC	NM_001192301.2
	R: GACCACTGAGGTAAGAGCCATC	
*c-MYC*	F: ATGGAGTGCCAGGCTCAAAG	BC113343.1
	R: CCGAATCGTAGTCGAGGTCATAG	
*HDAC1*	F: GCTGGCAAAGGCAAGTATTATC	NM_001037444.2
	R: CAGCATCAGCATAGGCAGGTT	
*BCL-2*	F: GCCCTGTGGATGACCGAGTA	NM_001166486.1
	R: GACAGCCAGGAGAAATCAAACA	
*GAPDH*	F:ATCTCGCTCCTGGAAGATG	BC102589.1
	R:TCGGAGTGAACGGATTCG	

F, forward; R, reverse.

*The reverse primer for qPCR experiments of miRNAs is a universal primer provided by the miScript II RT Kit (Qiagen).

### Nuclear Transfer, Fusion, Activation, and Culture of Cloned Embryos

Nuclear transfer, fusion, activation, and culture of embryos were performed according to the previous description^45^. Briefly, the COCs were treated with 0.1% bovine testicular hyaluronidase to discard the cumulus cells, and oocytes with the first polar (metaphaseII) were selected for further enucleation, which processed with a 20 μm (internal diameter) glass pipette by aspirating the first polar body and a small amount of surrounding cytoplasm in microdrops of PBS supplemented with 7.5 μg/ml cytochalasin B (CB) and 10% FBS. The cells of the NT-miR-449b group (dox-induced) and NT-control group (no dox-induced) were used as donor cells for nuclear transfer. The oocyte-cell couplet was sandwiched between a pair of platinum electrodes connected to a micromanipulator in microdrops of Zimmermann’s fusion medium, and a double electrical pulse of 35 V for 10 msec was applied for fusion^46^. Reconstructed embryos were kept in modified synthetic oviductal fluid (mSOF) supplementing with 5 mg/mL CB for 2 h^47^. Then, all reconstructed embryos were treated by 5 μM ionomycin for 4 min, followed by 1.9 mM dimethylaminopyridine for 4 h. After activation, the reconstructed embryos were cultured in G1.5 medium (Vitrolife AB, Gothenburg, Sweden) with mineral oil. We defined the day when reconstructed embryos were cultured as day 0, and the G2.5 medium was replaced on the third day of culture.

### *In vitro* fertilization

IVF were carried out as previous description^44^. Briefly, COCs with 3 layers of cumulus cell and uniform cytoplasm were selected and transferred into 200 μL microdrop of BO medium covered by mineral oil. Then, we added 2 × 10^6^ spermatozoa/mL of sperm suspension into the microdrop and incubated in a humid atmosphere of 5% CO_2_ at 38.5 °C for 20 h. After fertilization was completed, the zygotes were washed twice in mSOF to remove the cumulus cells and redundant sperm. Finally, the presumptive zygotes were cultured in 400 μL of G1 medium under mineral oil. We defined the day when IVF embryos were cultured as the first day and recorded subsequent development of embryos.

### Immunofluorescence staining of embryos

Embryos at different development stages (2-cell, 8-cell, and blastocyst) in the IVF group, NT-miR-449b group and NT-control group were collected for immunofluorescence staining, respectively. The method of immunofluorescence staining was based on our previous description^48^. After washings with PBS–polyvinyl alcohol (PVA), all embryos were fixed for 30 min in Immounol Staining Fix Solution (Beyotime, China) followed by permeabilizing for 30 min with PBS-PVA supplemented 0.2% Triton X-100. Then, embryos were incubated in Immounol Staining Blocking Solution (Beyotime) at 4 °C for 12 h followed by incubating overnight in the first antibodies to H3K9 acetylation (dilution concentration 1:1000, ab10812, Abcam, Cambridge, UK) at 4 °C. The seconeary antibodies for H3K9 acetylation (Alexa Fluor 488-labeled goat anti-rabbit IgG, dilution concentration 1:500, A0423,Beyotime) was added for incubating at room temperature for 2 h after three washings with PBS-PVA. The DNA was stained with 4′,6-diamidino-2-phenylindole (DAPI, C1005, Beyotime) for 3–5 min. Finally, glass slides with embyros were made and analysised using the Nikon Eclipse Ti-S microscope equipped with a 198 Nikon DS-Ri1 digital camera (Nikon, Tokyo, Japan).

The intensity of H3K9ac was analyzed using Image-Pro Plus software (Version 6.0; Media Cybernetics Corporation) and compared with that of DAPI signal. To quantify the fluorescent intensity of the H3K9ac in embryos from different groups, uniform negative and printing exposure time were used.

### Apoptosis assays

The DeadEnd Fluorometic TUNEL System (Promega) was used to process the apoptosis assays of blastocysts as the described of our previous study^48^. The treatment of the embryo before permeabilizing was same as that of immunofluorescence staining. After that, blastocysts were transferred into equilibration buffer for 5 min, followed by 1 h at 37 °C incubated with rTdT incubation buffer in the dark. Then, all samples were treated in 2 × SSC for 15 min to terminate the tailing reaction and stained the DNA with DAPI. Finally, glass slides with embryos were made and analyzed using the Nikon Eclipse Ti-S microscope equipped with a 198 Nikon DS-Ri1 digital camera (Nikon).

### Statistical Analysis

Experiments were repeated at least thrice, and each replicate was performed using oocytes matured on the same day to remove any batch effect of oocytes. All embryos were allocated randomly to each treatment group. The cleavage rate, blastocyst formation rate and apoptosis index were analyzed usingone-way ANOVA. The relative abundance of gene transcripts were established by testing the data for normal and equal variance using the Levene median test, ANOVA, and followed multiple pair wise comparisons using the Tukey’s test. The two-tailed Student’s t-test was used for pairwise comparisons of relative luciferase activity. Statistical analyses were conducted using the SPSS 22.0 software package (SPSS Inc., Chicago, IL, USA). Data were expressed as mean ± standard errors of the mean (SEM) and P < 0.05 was considered statistically significant.

## Electronic supplementary material

Supplementary Information
