# Supplementary material for: Sperm-borne miR-449b influences cleavage, epigenetic reprogramming and apoptosis of SCNT embryos in bovine
Source: Sci Rep. 2017 Oct 17;7:13403. doi: 10.1038/s41598-017-13899-8 (PMC5645405; doi:10.1038/s41598-017-13899-8)
Supplement: Supplementary file 1 — Supplementary Information [file 41598_2017_13899_MOESM1_ESM.pdf]

Sperm-borne miR-449b influences cleavage, epigenetic reprogramming and apoptosis of SCNT embryos in bovine

Mengyun Wang[a], Yang Gao[b], Pengxiang Qu[a],Suzhu Qing[a], Fang Qiao[a], Yong Zhang[a*], Jesse Mager[c*], Yongsheng Wang[a*]

a. College of Veterinary Medicine, Northwest A&F University, Key Laboratory of Animal Biotechnology of the Ministry of Agriculture, Yangling, Shaanxi Province 712100, PR China

b. Maternity & Child Health Care Hospital, Baoji, Shaanxi Province, 721000, PR China

c. Department of Veterinary and Animal Sciences, University of Massachusetts, Amherst, MA 01003, USA

Supplementary Fig. S1
Supplementary Fig. S2
Supplementary Fig. S3

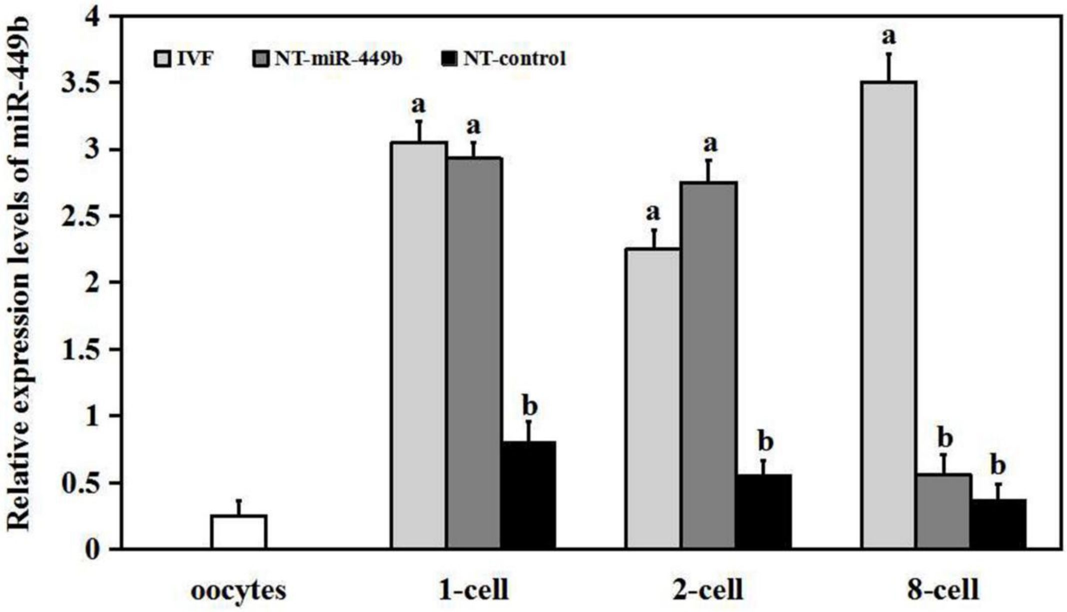

Supplementary Fig. S1 Quantitative (q) PCR analysis of relative expression levels of miR-449b in bovine oocytes as well as embryos derived from IVF, NT-control and NT-miR449b group, respectively. miR-449b expression was similar in IVF and NT-miR449b and significantly higher than NT-control at the 1-cell stage, then the level decreased, especially in NT-control. However, the relative expression level of miR-449b significantly increased in IVF group at the 8-cell stage. The bars graphs indicate the mean ± SEM from three independent biological samples. a, b values with different superscripts within columns are significantly different from each other ($P < 0.05$).

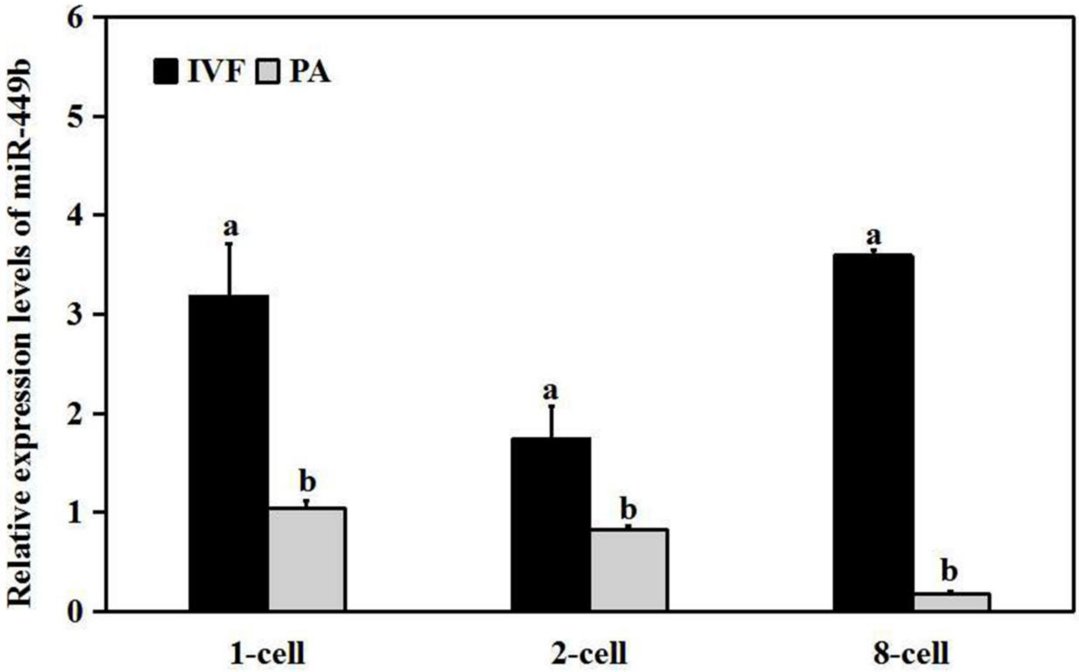

Supplementary Fig. S2 Quantitative (q) PCR analysis of relative expression levels of miR-449b in bovine embryos derived from IVF and parthenogenetic activation (PA). As shown in the result, miR-449b was more strongly expressed in IVF at early development stages compared to PA embryos. The bars graphs indicate the mean ± SEM from three independent biological samples. a, b values with different superscripts within columns are significantly different from each other ($P < 0.05$).

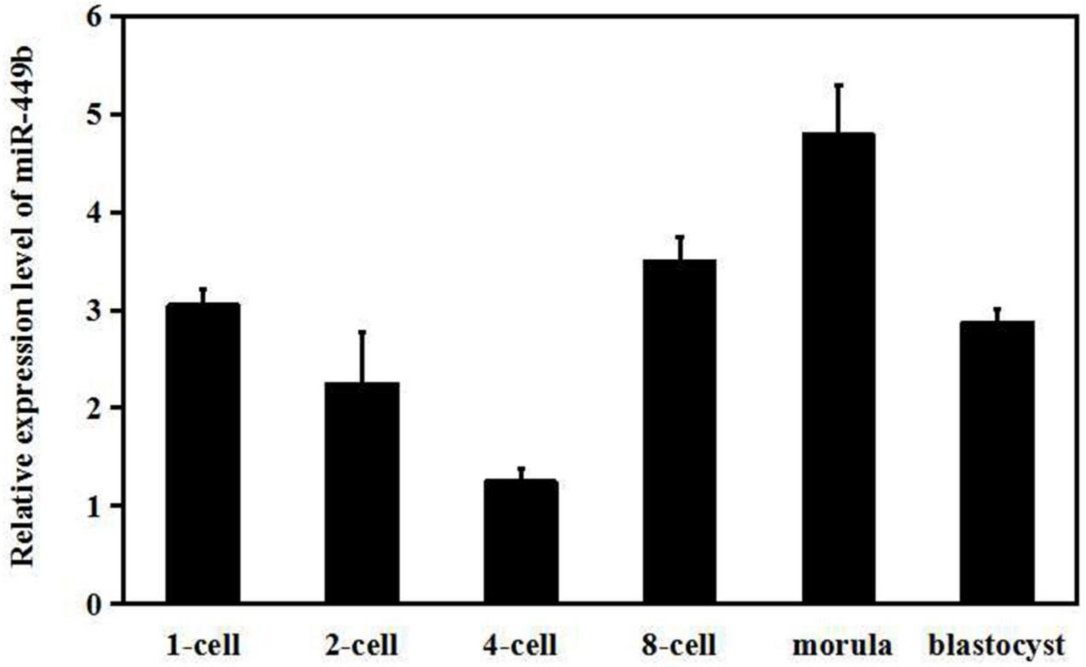

Supplementary Fig. S3 The expression pattern of miRNA-449b in bovine IVF preimplantation embryos. As shown in the result, miR-449b expression decreased at the 1-cell to 4-cell stage, then increased in the 8-cell and morula stage. When developed to blastocyst, the level of miR-449b decreased again, but still higher than that in 4-cell stage.
